# Evaluation of stimulus-effect relations in left ventricular growth using a simple multiscale model

**DOI:** 10.1007/s10237-019-01209-2

**Published:** 2019-08-06

**Authors:** Emanuele Rondanina, Peter H. M. Bovendeerd

**Affiliations:** Eindhoven University of Technology, PO Box 513, 5600 MB Eindhoven, The Netherlands

**Keywords:** Left ventricle, Concentric growth, Eccentric growth, Aortic stenosis, Aortic regurgitation, Mitral regurgitation

## Abstract

Cardiac growth is the natural capability of the heart to change size in response to changes in blood flow demand of the growing body. Cardiac diseases can trigger the same process leading to an abnormal type of growth. Prediction of cardiac growth would be clinically valuable, but so far published models on cardiac growth differ with respect to the stimulus-effect relation and constraints used for maximum growth. In this study, we use a zero-dimensional, multiscale model of the left ventricle to evaluate cardiac growth in response to three valve diseases, aortic and mitral regurgitation along with aortic stenosis. We investigate how different combinations of stress- and strain-based stimuli affect growth in terms of cavity volume and wall volume and hemodynamic performance. All of our simulations are able to reach a converged state without any growth constraint, with the most promising results obtained while considering at least one stress-based stimulus. With this study, we demonstrate how a simple model of left ventricular mechanics can be used to have a first evaluation on a designed growth law.

## Introduction

Cardiac growth is a natural process through which the heart adapts to deal with a change in blood flow demand of the body, which can be related either to changes in physical exercise (Rawlins et al. 2009) or cardiovascular diseases. It has been demonstrated how pressure overload promotes thickening of the cardiac wall, defined as concentric type of growth, while volume overload generates a dilated heart with a thinning of the wall, defined as eccentric growth (Cantor et al. 2005). Abnormal growth can alter also fetal hearts as has been discussed in Dewan et al. (2017) for the hypoplastic left heart syndrome.

**Fig. 1:**
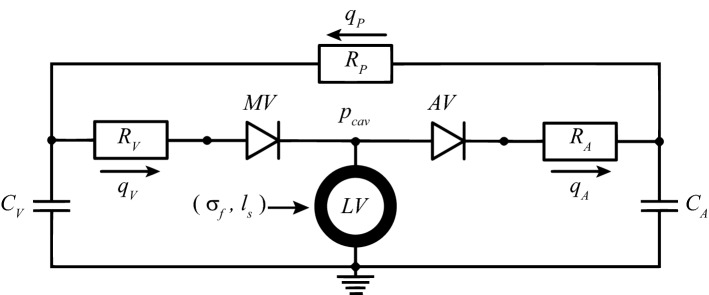
Lumped parameter model of the circulation. With mitral valve (MV), aortic valve (AV), venous and arterial resistance ($R_{\text {V}}$ and $R_{\text {A}}$) and capacitance ($C_{\text {V}}$ and $C_{\text {A}}$), peripheral resistance ($R_{\text {P}}$) and venous, arterial and peripheral flows ($q_{\text {V}}$, $q_{\text {A}}$, $q_{\text {P}}$). This model is coupled with the one-fiber model of LV mechanics

**Fig. 2:**
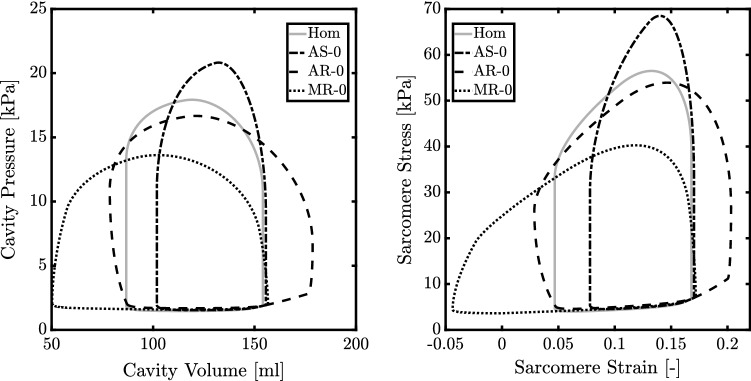
Pressure–volume (left) loop and sarcomere stress–strain (right) loop for the normal heart (Hom) and hearts with aortic stenosis (AS-0), aortic regurgitation (AR-0) and mitral regurgitation (MR-0) without growth

**Fig. 3:**
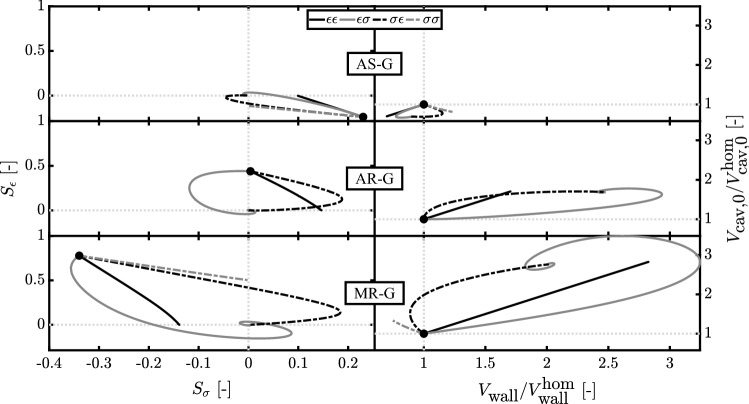
Evolution of stress-based $S_{\sigma }$ and strain-based $S_{\epsilon }$ stimuli (left) along with the ratio of wall volume $V_{\text {wall}}$ and cavity volume at zero pressure $V_{\text {cav,0}}$ in respect with their starting values at the homeostatic state, $V_{\text {wall}}^{\text {hom}}$ and $V_{\text {cav,0}}^{\text {hom}}$, respectively (right). Results are related to aortic stenosis (AS-G), aortic regurgitation (AR-G) and mitral regurgitation (MR-G) for strain- ($\epsilon$) and stress- ($\sigma$) based stimuli acting on $V_{\text {wall}}$ (first index) and $V_{\text {cav,0}}$ (second index). The starting point after inducing the valve pathology but before growth is identified by a dot

Cardiac growth is a complex mechanism involving many sub-processes which occur at different scales, from tissue level (cellular hypertrophy, apoptosis, proliferation, extracellular matrix remodeling) (Weber and Brilla 1991) to organ level (shape and dimensions) (Smith and Bishop 1985; Cheng et al. 2006). So far several models (Bovendeerd 2012; Witzenburg and Holmes 2017) have been published describing cardiac growth at both levels; however, the related driving force, namely the growth stimulus, is still under debate. While some studies used only one stimulus (Arts et al. 2005; Kroon et al. 2009), others distinguished between multiple stimuli, derived from multiple directions in the tissue or from different instants during the cardiac cycle, working simultaneously (Kerckhoffs et al. 2012; Taber 1998) or being pre-selected depending on the type of simulation (Göktepe et al. 2010).

**Fig. 4:**
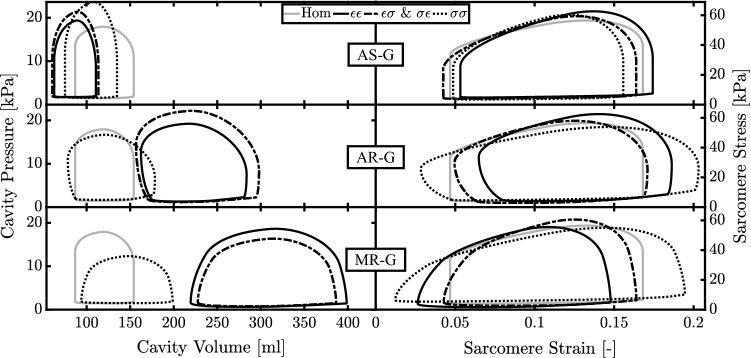
Cavity pressure–volume loops and the sarcomere stress–strain loops after growth during aortic stenosis (AS-G), aortic regurgitation (AR-G) and mitral regurgitation (MR-G) for strain- ($\epsilon$) and stress- ($\sigma$) based stimuli acting on wall volume (first index) and cavity volume (second index). All the simulations with $\epsilon \epsilon$ and $\sigma \epsilon$ as stimuli have overlapped loops

**Fig. 5:**
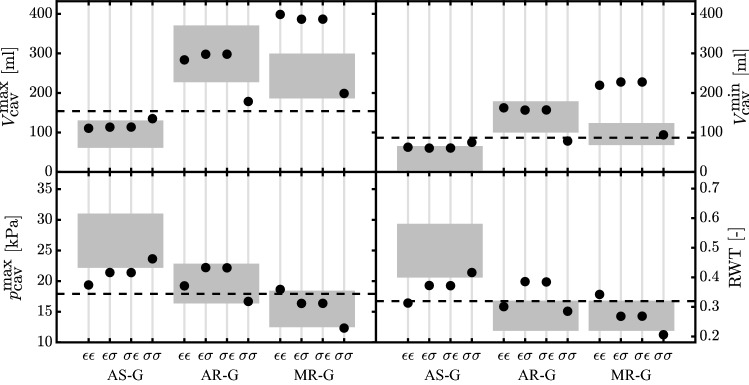
Maximum and minimum LV cavity volume ($V_{\text {cav}}^{\text {max}}$ and $V_{\text {cav}}^{\text {min}}$) along with maximum systolic pressure ($p_{\text {cav}}^{\text {max}}$) and relative wall thickness (RWT) after growth for aortic stenosis (AS-G), aortic regurgitation (AR-G) and mitral regurgitation (MR-G). The simulations are labeled according to their stimuli combinations: $\epsilon \epsilon$ has the strain stimulus on $V_{\text {wall}}$ and $V_{\text {cav,0}}$; $\sigma \sigma$ has the stress stimulus on $V_{\text {cav,0}}$ and $V_{\text {wall}}$; $\sigma \epsilon$ has the stress stimulus on $V_{\text {wall}}$ and strain stimulus on $V_{\text {cav,0}}$; $\epsilon \sigma$ has the strain stimulus on $V_{\text {wall}}$ and stress stimulus on $V_{\text {cav,0}}$. The model results are compared with patient data (gray boxes) (Carroll et al. 1992; Guzzetti et al. 2019; Kainuma et al. 2011; Seldrum et al. 2018; Villari et al. 1992). The dashed lines identify the homeostatic level of the model

**Fig. 6:**
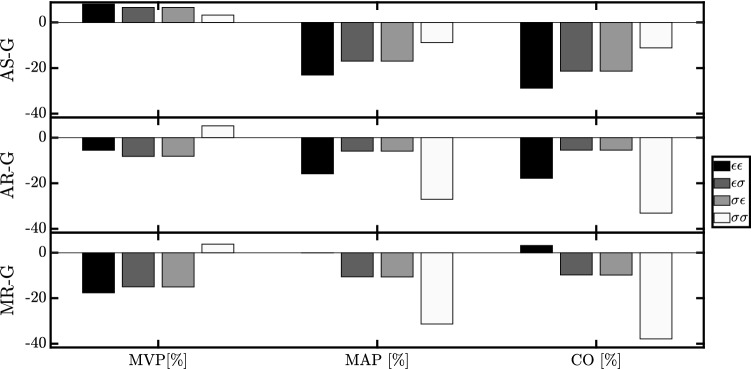
Hemodynamic change in terms of mean venous pressure (MVP), mean arterial pressure (MAP) and cardiac output (CO) in respect with the homeostatic state. Results are shown for aortic stenosis (AS-G), aortic regurgitation (AR-G) and mitral regurgitation (MR-G) for strain- ($\epsilon$) and stress- ($\sigma$) based stimuli acting on wall volume (first index) and cavity volume (second index)

A second point of debate is whether or not to constrain maximum growth. In the literature, we find both unconstrained growth laws (Bovendeerd et al. 2006; Kroon et al. 2009; Taber 1998) and constrained laws, the latter being motivated from experimental observations on myocyte properties (Göktepe et al. 2010; Kerckhoffs et al. 2012; Rausch et al. 2011) as well as availability of nutrients (Bellomo et al. 2012).

The aim of this study is to evaluate the capability of several combinations of growth stimuli, based on myocardial tissue stress and strain, to reproduce a clinically realistic and stable changes of left ventricular (LV) geometry, i.e., LV cavity volume and wall volume, in response to changes in LV hemodynamic load.

We use one single law in which we do not constrain maximum growth or prescribe the type of growth, concentric or eccentric, a priori.

We combine this law with a lumped parameter model of cardiovascular mechanics, focusing on growth induced by valve diseases. Specifically we consider aortic stenosis (AS), in which the increase in valve resistance leads to concentric growth (Buckert et al. 2018; Guzzetti et al. 2019; Lamb et al. 2002), along with aortic regurgitation (AR) and mitral regurgitation (MR), which lead to an eccentric growth due to an increase in blood flow into the ventricular cavity (Gaasch et al. 1983; Kleaveland et al. 1988; Lamb et al. 2002; Nakano et al. 1991; Wisenbaugh et al. 1984; Zile et al. 1991). Although MR and AR are characterized by volume overload, the latter is described by a higher systolic pressure (Kusunose et al. 2015; Seldrum et al. 2018; Wisenbaugh et al. 1984; Zile et al. 1985). For this reason, we study both diseases.

## Methods

Our model of cardiac growth is based on the interaction between a model coupling the LV mechanics at the tissue level and the organ level, with a model for the hemodynamics in the systemic circulation, and a growth model in which LV wall ($V_{\text {wall}}$) and cavity ($V_{\text {cav}}$) volumes respond to deviations of actual tissue load from the corresponding homeostatic value.

### LV mechanics model

We use the one-fiber model of cardiac function (Arts et al. 1991; Bovendeerd et al. 2006) to relate mechanics at the organ level, expressed in terms of left ventricular pressure $p_{\text {cav}}$ and volume $V_{\text {cav}}$, to mechanics at the tissue level, expressed with myofiber stress $\sigma _{\text {f}}$ and sarcomere length $l_{\text {s}}$. The main equations for LV mechanics are:

1a $$\begin{aligned} p_{\text {cav}} \;&=\; \frac{1}{3} \; (\sigma _{\text {f}}) \; \text {ln}\left( 1 \;+\; \frac{V_{\text {wall}}}{V_{\text {cav}}} \right) \end{aligned}$$

1b $$\begin{aligned} \lambda _{\text {f}} \;&=\; \frac{l_{\text {s}}}{l_{\text {s,0}}} \;=\; \left( \frac{V_{\text {cav}} \;+\; \frac{1}{3} \; V_{\text {wall}}}{V_{\text {cav,0}} \;+\; \frac{1}{3} \; V_{\text {wall}}} \right) ^{\frac{1}{3}} \end{aligned}$$

Here $V_{\text {cav,0}}$ is the cavity volume in the unloaded state and $\lambda _{\text {f}}$ is the fiber stretch ratio. Myofiber stress $\sigma _{\text {f}}$ is composed of an active component $\sigma _{\text {a}}$ and two passive components, generated by the collagen matrix along-fiber direction $\sigma _{\text {m,f}}$ and radial direction $\sigma _{\text {m,r}}$. As consequence $\sigma _{\text {f}}$ is defined as follows:

2 $$\begin{aligned} \sigma _{\text {f}} \;=\; \sigma _{\text {a}}(l_{\text {s}},\, t_{\text {a}},\, v_{\text {s}}) \;+\; \sigma _{\text {m,f}}(\lambda _{\text {f}}) \;-\; 2\sigma _{\text {m,r}}(\lambda _{\text {r}}) \end{aligned}$$

where $v_{\text {s}}$ is the sarcomere shortening velocity, $t_{\text {a}}$ is the time elapsed since activation and $\lambda _{\text {r}}$ is the resulting tissue stretch in radial direction under the assumption of incompressibility. The functional form for $\sigma _{\text {m,f}}$ and $\sigma _{\text {m,r}}$ was taken from Bovendeerd et al. (2006), while $\sigma _{\text {a}}$ was adopted from van der Hout-van et al. (2012). More details on these constitutive laws can be found in Appendix.

### Systemic circulation model

At the organ level, $p_{\text {cav}}$ and $V_{\text {cav}}$ are determined from the interaction between the LV and the systemic circulation (Fig. 1). Arteries (A), veins (V) and peripheral vessels (P) are modeled by capacitance *C* and resistances *R*, with a resulting pressure drop over the capacitance $\varDelta p_{\text {c}}$ and the resistance $\varDelta p_{\text {r}}$ defined as follows:

3 $$\begin{aligned} \varDelta p_{\text {r}} \;=\; R q_{\text {r}}; \qquad \varDelta p_{\text {c}} \;=\; \frac{V_{\text {c}} - V_{\text {c,0}}}{C} \end{aligned}$$

Here $q_{\text {r}}$ is the flow through the resistance, $V_{\text {c}}$ is the volume in the capacitance and $V_{\text {c,0}}$ is the same volume at zero pressure. The flow through aortic valve ($q_{\text {A}}$) and mitral valve ($q_{\text {V}}$) is determined by the corresponding *R*, combined with a dimensionless resistance parameter *k*, which is set to $k_{\text {f}}$ for forward flow and $k_{\text {b}}$ for backward flow:

4 $$\begin{aligned} q_{\text {(A;V)}} \;=\; \frac{\varDelta p_{\text {(A;V)}}}{kR_{\text {(A;V)}}} \;\text {with}\; {\left\{ \begin{array}{ll} \; k \;=\; k_{\text {f}} & \text {for} \;\varDelta p_{\text {(A;V)}} > 0 \\ \; k \;=\; k_{\text {b}} & \text {for} \;\varDelta p_{\text {(A;V)}} \le 0 \end{array}\right. } \end{aligned}$$

A healthy valve is represented by $k_{\text {f}}$ equal to 1 and $k_{\text {b}}$ equal to $10^{6}$.

**Fig. 7:**
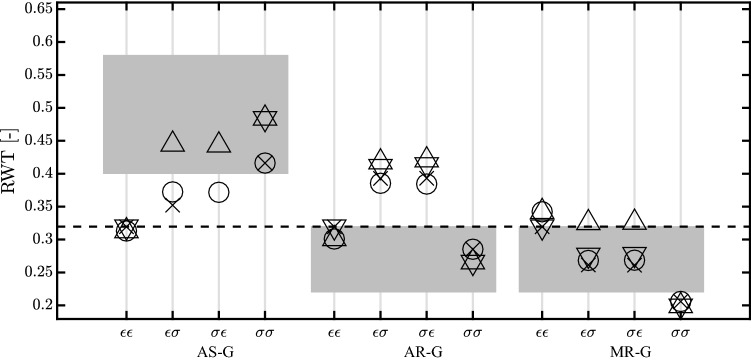
Relative wall thickness (RWT) after growth for aortic stenosis (AS-G), aortic regurgitation (AR-G) and mitral regurgitation (MR-G). The dashed line identifies the homeostatic level of the model. Each symbol ( $\bigcirc$, $\times$, $\bigtriangleup$ and $\bigtriangledown$ ) refers to a different growth stimulus. We used mean stress combined with sarcomere strain amplitude ($\bigcirc$) and maximum strain ($\times$), peak systolic stress combined with sarcomere strain amplitude ($\bigtriangleup$) and maximum strain ($\bigtriangledown$). All the stimuli combinations on $V_{\text {wall}}$ and $V_{\text {cav,0}}$ are labeled as follows : $\epsilon \epsilon$ has the strain stimulus on $V_{\text {wall}}$ and $V_{\text {cav,0}}$; $\sigma \sigma$ has the stress stimulus on $V_{\text {cav,0}}$ and $V_{\text {wall}}$; $\sigma \epsilon$ has the stress stimulus on $V_{\text {wall}}$ and strain stimulus on $V_{\text {cav,0}}$; $\epsilon \sigma$ has the strain stimulus on $V_{\text {wall}}$ and stress stimulus on $V_{\text {cav,0}}$. Three combinations did not reach a stable ending state for AS-G and hence are not present in the picture. The combinations are $\epsilon \sigma$ for $\bigtriangledown$, $\sigma \epsilon$ for $\times$ and $\bigtriangledown$

**Table 1:** List of parameters used in the model

Tissue			Organ		
Parameter	Value	Unit	Parameter	Value	Unit
$a_{\text {d}}$	400	ms/$\upmu$m	$C_{\text {A}}$	20	ml kPa
$a_{\text {r}}$	100	ms/$\upmu$m	$C_{\text {V}}$	600	ml kPa
$c_{\text {a}}$	1.2	$\mu \hbox {m}^{-1}$	$R_{\text {A}}$	10	kPa ms/ml
$c_{\text {f}}$	11.7	–	$R_{\text {P}}$	120	kPa ms/ml
$c_{\text {r}}$	9	–	$R_{\text {V}}$	1	kPa ms/ml
$l_{\text {sa,0}}$	1.5	$\upmu$m	$T_{\text {cyc}}$	800	ms
$l_{\text {sa,1}}$	2	$\upmu$m	$V_{\text {A,0}}$	500	ml
$\sigma _{\text {a,0}}$	150	kPa	$V_{\text {blood}}$	5000	ml
$\sigma _{\text {f,0}}$	0.9	kPa	$V_{\text {cav,0}}$	67	ml
$\sigma _{\text {r,0}}$	0.2	kPa	$V_{\text {V,0}}$	3000	ml
$\tau _{\text {d,1}}$	250	ms	$V_{\text {wall}}$	200	ml
$\tau _{\text {r,1}}$	150	ms	$k_{\text {f}}$	1	–
$v_{\text {0}}$	0.01	ms/$\upmu$m	$k_{\text {b}}$	$10^{6}$	–

The chosen values are adapted from (van der Hout-van et al. 2012). Valve parameters $k_{\text {f}}$ and $k_{\text {b}}$ are modified for every valve disease. For aortic stenosis $k_{\text {f}}$ is increased to 3, for aortic regurgitation $k_{\text {b}}$ is decreased to 6 while for mitral regurgitation $k_{\text {b}}$ is decreased to 30

### Growth model

To control cardiac growth, we apply stress- or strain-related measures. As the stress-related measure $L_{\sigma }$ we consider the mean of the fiber stress $\sigma _{\text {f}}$ (Eq. 2) over a complete cardiac cycle of length $T_{\text {cyc}}$:

5 $$\begin{aligned} L_{\sigma } \;=\; \frac{1}{T_{\text {cyc}}} \; \int _0^{T_{\text {cyc}}} \! \sigma _{\text {f}}(t) \; {\mathrm {d}}t \end{aligned}$$

As the strain-related measure $L_{\epsilon }$ we use the sarcomere strain amplitude during the same cycle:

6 $$\begin{aligned} L_{\epsilon } \;=\; \text {max}(\epsilon ) \;-\; \text {min}(\epsilon ) \quad \text {with} \quad \epsilon = \text {ln} \left( \lambda _{\text {f}}\right) \end{aligned}$$

Consequently, we define stress-based $S_{\sigma }$ and strain-based $S_{\sigma }$ stimuli for growth as follows:

7 $$\begin{aligned} S_{\sigma } \;=\; \frac{L_{\sigma } \;-\; L_{\sigma ,\text {hom}}}{L_{\sigma ,\text {hom}}} \;; \qquad S_{\epsilon } \;=\; \frac{L_{\epsilon } \;-\; L_{\epsilon ,\text {hom}}}{L_{\epsilon ,\text {hom}}} \end{aligned}$$

in which $L_{\sigma ,\text {hom}}$ and $L_{\epsilon ,\text {hom}}$ represents the homeostatic tissue load for $L_{\sigma }$ and $L_{\epsilon }$, respectively.

At the tissue level, growth can be either in the direction of the myofibers or perpendicular to their orientation. Considering the organization of the myofibers in the cardiac wall, along-fiber and cross-fiber growth at the tissue level correspond to an increase of $V_{\text {cav,0}}$ and $V_{\text {wall}}$ at the organ level, respectively. In view of the governing equations for the one-fiber model (Eq. 1), we model the response to a stress-based stimulus as:

8a $$\begin{aligned} \frac{1}{V_{\text {wall}}} \; \frac{\text {d}V_{\text {wall}}}{\text {d}t}&\;=\; +\; \frac{S_{\sigma }}{\tau _{\text {grw}}} \end{aligned}$$

8b $$\begin{aligned} \frac{1}{V_{\text {cav,0}}} \; \frac{\text {d}V_{\text {cav,0}}}{\text {d}t}&\;=\; -\; \frac{S_{\sigma }}{\tau _{\text {grw}}} \end{aligned}$$

Similarly, the response to a strain-based stimulus is modeled as:

9a $$\begin{aligned} \frac{1}{V_{\text {wall}}} \; \frac{\text {d}V_{\text {wall}}}{\text {d}t}&\;=\; +\; \frac{S_{\epsilon }}{\tau _{\text {grw}}} \end{aligned}$$

9b $$\begin{aligned} \frac{1}{V_{\text {cav,0}}} \; \frac{\text {d}V_{\text {cav,0}}}{\text {d}t}&\;=\; +\; \frac{S_{\epsilon }}{\tau _{\text {grw}}} \end{aligned}$$

For both Eqs. 8 and 9, the growth rate constant is defined by $\tau _{\text {grw}}$.

### Simulations performed

Initial simulations were performed with parameter settings adopted from van der Hout-van et al. (2012), with some settings adapted slightly to achieve realistic function for a healthy human (Table 1). This healthy state was considered the normal homeostatic state (Hom), from which we collect values for $L_{\sigma ,\text {hom}}$ and $L_{\epsilon ,\text {hom}}$. Second, we simulated three types of valve disease. To simulate AS, we consider a threefold increase of $k_{\text {f}}$, based on experimental data (Roger et al. 1997). To simulate AR and MR, we lowered $k_{\text {b}}$ to 6 and 30, respectively, in order to obtain a regurgitant fraction, defined as the ratio between backward and forward volume, close to 0.6 for both MR (Kleaveland et al. 1988; Nakano et al. 1991) and AR (Wisenbaugh et al. 1984). These pathological cases are labeled AS-0, AR-0 and MR-0. Each perturbation altered pressure and volume at the organ level, causing tissue stress and strain to deviate from their homeostatic values (Fig. 2) and consequently leading to nonzero stimuli $S_{\sigma }$ and $S_{\epsilon }$. Third, these stimuli were used as input to simulate LV growth, resulting in changes of $V_{\text {cav,0}}$ and $V_{\text {wall}}$ according to Eqs. 8 and 9. We evaluated all possible combinations of these equations, although it might be more realistic for the myocytes to compensate both internal stress and strain instead of having one preferred loading measure. We labeled our simulations $\sigma$ for a stress-based (Eq. 8) and $\epsilon$ for a strain-based stimulus (Eq. 9). In the resulting label, the first letter indicates the growth stimulus used for $V_{\text {wall}}$, while the second refers to $V_{\text {cav,0}}$ stimulus. In specific $\epsilon \epsilon$ is given by Eqs. 9a and 9b; $\epsilon \sigma$ by Eqs. 9a and 8b; $\sigma \epsilon$ by Eqs. 8a and 9b and finally $\sigma \sigma$ by Eqs. 8a and 8b. The diseased grown state is identified with AS-G for AS, AR-G for AR and MR-G for MR. The constant $\tau _{\text {grw}}$ from Eqs. 8 and 9 is set to a value of $T_{\text {cyc}}/25$ while considering a time step $\varDelta t$ of 1 ms and a $T_{\text {cyc}}$ of 800 ms.

To characterize the obtained grown state, we use the relative wall thickness (RWT) defined as:

10 $$\begin{aligned} \text {RWT} \;=\; \frac{2H_{\text {wall}}}{D_{\text {cav}}} \end{aligned}$$

where $D_{\text {cav}}$ is the LV cavity diameter at the end diastole while $H_{\text {wall}}$ is the LV wall thickness. From Seldrum et al. (2018), the healthy RWT is identified with 0.31 ± 0.06. As a consequence, a higher RWT identifies a concentric type of growth while a lower value indicates an eccentric type of growth. In order to define $D_{\text {cav}}$ and $H_{\text {wall}}$, we approximate the LV shape as a thick-walled sphere.

## Results

The healthy LV has a stroke volume (SV) of approximately 67 ml, with highest volume $V_{\text {cav}}^{\text {max}}$ of 154 ml and lowest volume $V_{\text {cav}}^{\text {min}}$ of about 87 ml (Fig. 2). Cardiac output (CO) is about 5 l/min, maximum systolic pressure ($p_{\text {cav}}^{\text {max}}$) is 18 kPa, mean arterial (MAP) and venous (MVP) pressure are 12 kPa and 1.9 kPa, respectively. From this healthy heart simulation, we derive the homeostatic tissue load values for $L_{\sigma ,\text {hom}}$ (18 kPa) and $L_{\epsilon ,\text {hom}}$ (0.12). Figure 2 also shows the effects of valve pathologies on pump and tissue function. For AS-0, AR-0 and MR-0, $p_{\text {cav}}^{\text {max}}$ changes by + 16%, $-\,7\%$ and $-\,24\%$, respectively, while SV range changes by $-\,20\%$, + 48% and + 58%. Characteristic tissue stress $L_{\sigma }$ changes by + 23% and $-\,34\%$ for AS-0 and MR-0, but remains unchanged for AR-0. Characteristic tissue strain $L_{\epsilon }$ changes by + 44%, + 78%, and $-\,24\%$ for AR-0, MR-0 and AS-0.

According to the growth model, the deviation of $$L_{\epsilon }$$ and $$L_{\sigma }$$ from their homeostatic values results in changes in cavity and wall volume. Figure 3 shows the evolution of $$S_{\epsilon }$$, $$S_{\sigma },$$$$V_{\text {cav,0}}$$ and $$V_{\text {wall}}$$ during growth. A stable ending state was found for all combinations of stimuli. Controlling both $$S_{\epsilon }$$ and $$S_{\sigma }$$, in simulations $$\epsilon \sigma$$ and $$\sigma \epsilon$$, leads to the same ending state independently by their application on $$V_{\text {cav,0}}$$ or $$V_{\text {wall}}$$. However, the temporal evolution of stimuli and volumes toward this ending state is different. Simulations $$\epsilon \epsilon$$ and $$\sigma \sigma$$ are able to restore either $$S_{\epsilon }$$ or $$S_{\sigma }$$, respectively, since their growth stimulus depends only on $$L_{\epsilon }$$ or $$L_{\sigma }$$. In general, trajectories of the stimuli and the volumes are straight in simulations $$\epsilon \epsilon$$ and $$\sigma \sigma$$, but curved in simulations $$\epsilon \sigma$$ and $$\sigma \epsilon$$. Aortic valve stenosis, AS-0, causes a change of the growth stimuli $$S_{\epsilon } \approx$$$$-\,0.2$$ and $$S_{\sigma } \approx +\,0.2$$. The final grown state AS-G is characterized by a decrease in both $$V_{\text {cav,0}}$$ and $$V_{\text {wall}}$$ in simulations $$\epsilon \epsilon$$, $$\epsilon \sigma$$ and $$\sigma \epsilon$$. In simulation $$\sigma \sigma$$, $$V_{\text {cav,0}}$$ also decreases but $$V_{\text {wall}}$$ increases. Aortic valve regurgitation, AR-0, causes stimuli $$S_{\epsilon } \approx$$ + 0.5 and a $$S_{\sigma } \approx$$ 0. In simulation $$\epsilon \epsilon$$, $$V_{\text {cav,0}}$$ and $$V_{\text {wall}}$$ both increase by about 70%. In simulations $$\epsilon \sigma$$ and $$\sigma \epsilon$$, the change in $$V_{\text {wall}}$$ is about twice as high. Volumes do not change in simulation $$\sigma \sigma$$, because $$S_{\sigma }$$ is zero. Mitral valve regurgitation, MR-0, yields the largest stimuli of the three cases, with $$S_{\epsilon } \approx$$ 0.8 and $$S_{\sigma } \approx -\,0.3$$. For MR-G simulation $$\epsilon \epsilon$$ results in an increase in cavity and wall volume by about 200%. In simulations $$\epsilon \sigma$$ and $$\sigma \epsilon$$, cavity volume increases by about the same amount, but wall volume increase is about 100%. In simulation $$\sigma \sigma$$, changes are much smaller, with an increase in cavity volume by about 30% and a decrease in wall volume of about 75%. Figure 4 shows organ function and tissue function after growth has been completed. AS-G is characterized by a decrease in the cavity volume followed by a decrease in SV of about 20% and an increase in $$p_{\text {cav}}^{\text {max}}$$ for all stimuli. At the tissue level, stress–strain loops after growth are similar to the healthy state, irrespective of the used stimulus combination. For AR-G simulations $$\epsilon \sigma$$ and $$\sigma \epsilon$$ have the highest increase in SV and pressure (+ 110% and + 23%, respectively). Simulation $$\epsilon \epsilon$$ and $$\sigma \sigma$$ follow the same trend for SV (+ 80% and + 50%, respectively) while having a $$p_{\text {cav}}^{\text {max}}$$ close to the homeostatic value ($$+7\%$$ for $$\epsilon \epsilon$$ and $$-7\%$$ for $$\sigma \sigma$$). Moreover, $$\epsilon \sigma , \sigma \epsilon$$ and $$\epsilon \epsilon$$ are all characterized by a similar increase in cavity volume. At the tissue level only $$\epsilon \sigma$$ and $$\sigma \epsilon$$ show a stress–strain loop similar to the healthy state.In MR-G, at the organ level, simulations $$\epsilon \sigma$$, $$\sigma \epsilon$$ and $$\epsilon \epsilon$$ follow a similar trend with an increase in cavity volumes and SV, which is higher than in AR-G, along with a smaller increase in $$p_{\text {cav}}^{\text {max}}$$. Only simulation $$\sigma \sigma$$ is characterized by a decrease in pressure of about 30%. At the tissue level $$\epsilon \sigma$$ and $$\sigma \epsilon$$ have a stress–strain loop close to the homeostatic state while $$\sigma \sigma$$ has the highest excursion in sarcomere strain.

With Fig. 5, we compare the model results with patient data for AS (Carroll et al. 1992; Guzzetti et al. 2019) and AR and MR (Kainuma et al. 2011; Seldrum et al. 2018; Villari et al. 1992). With the model parameters shown in Table 1, we obtain a homeostatic RWT of 0.32, which is in line with Seldrum et al. (2018). For AS-G, all the stimuli combinations have an ending state in accordance with the clinical trend. More specifically, the volumes for $\epsilon \epsilon$, $\epsilon \sigma$ and $\sigma \epsilon$ are in the observed range, while only $\sigma \sigma$ is able to predict a $p_{\text {cav}}^{\text {max}}$ and RWT in accordance with patient data. With AR-G only $\sigma \sigma$ is characterized by lower volumes than patient data; however, all the stimuli predict a correct $p_{\text {cav}}^{\text {max}}$. The change in RWT indicates a concentric growth for $\epsilon \sigma$ and $\sigma \epsilon$ but a slight eccentric growth for $\epsilon \epsilon$ and $\sigma \sigma$. Only the last two are in line with clinical findings. Eventually with MR-G all the volumes have the same trend of patient data but only $\sigma \sigma$ is in the observed range. By evaluating $p_{\text {cav}}^{\text {max}}$ and RWT, simulations $\epsilon \sigma$ and $\sigma \epsilon$ yield results in the clinical range, while results for simulations $\sigma \sigma$ and $\epsilon \epsilon$ are at the border of clinical data, with the latter being characterized by an opposite trend.

Figure 6 shows the evolution of hemodynamics with respect to the Hom simulation. For AS-G, all the stimuli combinations lead to an ending state characterized by an increase in MVP of about $+\,5\%$ along with a decrease in MAP between $-\,26\%$ and $-\,9\%$. CO is reduced in proportion to the decrease in the difference MAP-MVP. For AR-G, we find an opposite change in MVP, except for simulation $\sigma \sigma$, but again decreases in MAP and CO. For MR-G, MVP, MAP and CO decrease in all simulations, with the only exception of $\epsilon \epsilon$ which has a MAP and CO close to the homeostatic state.

## Discussion

Cardiac growth is one of the mechanisms through which the heart can respond to long-term changes of the body. Although the main characteristics of this process have been already defined in the literature, a numerical model capable of predicting a reliable and unconstrained disease progress is currently under debate. A clear relation between the growth driving force, defined as the stimulus, and the effect on tissue and organ level is still missing. Solving this challenge represents a crucial improvement in supporting clinical decision making.

The aim of this study is to evaluate the capability of several combinations of growth stimuli to reproduce a clinically realistic and stable changes of left ventricular (LV) geometry in response to changes in LV hemodynamic load. To achieve this goal, we tested several combinations of stress-based and strain-based stimuli in a simplified model of the LV mechanics (Bovendeerd et al. 2006). We stimulate growth in response to aortic valve stenosis (AS) and regurgitation (AR) and mitral valve regurgitation (MR). We describe cardiac growth with a single global law, appropriate for concentric and eccentric growth, unlike models which use different laws (Göktepe et al. 2010). Moreover, we prefer not to define an a priori stopping criterion, in contrast to Bellomo et al. (2012), Göktepe et al. (2010), Kerckhoffs et al. (2012), and Rausch et al. (2011). We assumed that in the real heart minor changes in cardiac load would lead to a stable new configuration, well within any possible limits for final growth. Thus the ability to reach such a stable situation would be an important criterion to evaluate a growth law. We use the one-fiber model to represent LV mechanics which provides a natural description of LV mechanics with a clear contribution of LV size and myocardial material properties. Consequently, changes in cavity and wall volume, as induced by growth, result in a change in LV function without a need to change tissue properties. This is an advantage in comparison with an even simpler LV mechanics model, the time-varying elastance model, in which a change in size has to be converted into a change in elastance, as demonstrated in a similar growth study by Witzenburg and Holmes (2018). In addition, the one-fiber model offers the possibility to use tissue level load, i.e., fiber stress and strain, as an input for growth. We approximate valve diseases by changing only a resistance for forward or backward flow.

We introduce growth with Eqs. 8 and 9, which sign is chosen to achieve stability. When running the model in Fig. 1, we observed how the mean stress (Eq. 5) decreased with the increase of $V_{\text {wall}}$ and decrease of $V_{\text {cav,0}}$. For this reason, a stress higher than the homeostatic level, resulting in a positive stress stimulus, was counteracted by an increase in $V_{\text {wall}}$ (positive sign in Eq. 8a ) and a decrease in $V_{\text {cav,0}}$ (negative sign in Eq. 8b). The same analysis showed how the sarcomere strain amplitude (Eq. 9) decreased with the increase of $V_{\text {cav,0}}$ as well as with an increase of $V_{\text {wall}}$. Thus, a positive strain stimulus was counteracted by an increase in $V_{\text {wall}}$ (positive sign in Eq. 9a) and $V_{\text {cav,0}}$ (positive sign in Eq. 9b). Growth is evaluated in terms of the relative wall thickness RWT. Simulation $\epsilon \epsilon$ is always characterized by a RWT close to the homeostatic value. This is a consequence of the stimulus-effect relation in Eq. 9, according to which a strain-based stimulus has a similar effect on cavity and wall volume. For $\sigma \sigma$, RWT deviates strongly from the homeostatic value. Indeed, according to stimulus-effect relation of Eq. 8, a stress-based stimulus has an opposite effect on cavity and wall volume. Finally, $\epsilon \sigma$ and $\sigma \epsilon$ are defined by opposite type of stimuli which follow different paths during cardiac growth (Fig. 3). Initially the volumes change oppositely for all the diseases. However, eventually the same ending state is achieved, indicating that there is only one configuration of $V_{\text {cav,0}}$ and $V_{\text {wall}}$ such that both stimuli are restored to their homeostatic values. Hence the choice of applying $S_{\sigma }$ and $S_{\epsilon }$ on $V_{\text {cav,0}}$ or $V_{\text {wall}}$ does not influence the final result of the model. As a consequence, we observe identical organ and tissue function loops in Fig. 4.

The growth stimuli are based on mean fiber stress and sarcomere strain amplitude during a cardiac cycle, see Eq. 5 and 6. We also investigated the influence of a different stress measure, peak systolic stress, and a different strain measure, maximum strain. Out of the twelve new combinations of stress and strain stimuli, nine combinations were found to lead to stable growth in all three pathologies (Fig. 7). The type of hypertrophy, however, was hardly affected in these cases. The remaining three combinations (mean stress for $V_{\text {wall}}$ with maximum strain for $V_{\text {cav,0}}$, identified with $\times$ symbol for $\sigma \epsilon$; peak systolic stress for $V_{\text {wall}}$ with maximum strain for $V_{\text {cav,0}}$, identified with $\bigtriangledown$ symbol for $\sigma \epsilon$; maximum strain for $V_{\text {wall}}$ with peak systolic stress for $V_{\text {cav,0}}$, identified with $\bigtriangledown$ symbol for $\epsilon \sigma$) yielded stable growth for the two regurgitation cases, but not for aortic stenosis. A more extensive evaluation of other stimuli was considered outside the scope of this paper. We also investigated the sensitivity of the model on the settings of the homeostatic load parameters. Changes of 20% were found to lead to a new converged state characterized by a similar change in $V_{\text {wall}}$ and $V_{\text {cav,0}}$ with a growth stimulus back to zero. Although it seems we have two growth processes for $V_{\text {wall}}$ and $V_{\text {cav,0}}$, they are actually closely linked at the tissue level. For this reason, we are using only one $\tau _{grw}$ for both volumes. With a series of simulations, we investigated the importance of this parameter observing that the final ending state was not influenced by a change of $\tau _{grw}$. Finally, we found that, upon removal of the pathology, cavity and wall volume evolved back to the values in the original homeostatic state.

In our study, we converted loading measures along myofiber direction into growth perpendicular to the fiber direction, changing $V_{\text {wall}}$, and along the fiber direction, changing $V_{\text {cav,0}}$. We always obtained a stable ending state. This observation is in line with the finding of Witzenburg and Holmes (2017). The authors gave a global view on the state of the art for growth models, demonstrating how only growth applied in multiple directions could recover the homeostatic states. Moreover, we showed how using only a stress-based, or only a strain-based stimulus can still describe the generic trend of pressure and volume overload with a converged state. This finding, however, does not correspond with Witzenburg and Holmes (2017), where it was concluded that at least two measures, poorly coupled during acute phase, were needed to achieve best results.

We compared the results of our growth simulations with clinical data taken from the literature. For AS, we used volumes and RWT from Guzzetti et al. (2019), which was based on a population of 93 patients, while for pressure values we used the work of Carroll et al. (1992), based on a population of 40 patients. For AR and MR, we took the clinical data from Seldrum et al. (2018) for both volumes and RWT. The number of patients having AR was 29 while for MR 59 patients were observed. Eventually we used two different studies to get LV systolic pressure values. For AR, we used Villari et al. (1992), with 30 patients, and Kainuma et al. (2011), having 46 patients. The collected data refer to patients having different degrees of severity of valve stenosis and regurgitation. Moreover, patients rarely suffer of an isolated valve pathology. However, in our study we investigated only one valve disease a time with only one degree of severity. Consequently, comparison between model results and the literature data can only be done qualitatively. In a similar study, Witzenburg and Holmes (2018) show how a better match between clinical and numerical data can be obtained by considering a customization of the hemodynamic parameters for each case. They not only tuned the valve pathology, but also adapted the systemic resistance and the stressed blood volume. Tuning our model to the individual patient as well would allow for a more strict test of the model. In particular, it would be interesting to see whether we could still use one homeostatic set point, derived from a generic healthy state, or whether the homeostatic set point should be set differently for different types of disease, as done by Witzenburg and Holmes (2018).

The proposed model, in its simplicity, lacks the spatial variability which can be offered by a finite elements (FE) approach. With a 3D FE model, both cross-fiber and along-fiber changes in stress and strain can be used as growth stimuli and growth might be defined locally along-fiber and cross-fiber, replacing our organ-level approach of growth of cavity and wall volume. In a FE model, it would also be possible to evaluate growth in response to spatially distributed changes in stimuli, as induced for example by localized infarctions or electrical conduction disorders. As a drawback, stability of model outcome would not only depend on the growth law, but also on the choice of boundary conditions and possible deterioration of element quality during growth (van Osta et al. 2019).

AS, AR and MR are taken as the testing ground for our model; however, we can consider as a valuable growth trigger every possible change in the preload and afterload of the LV. It is important to highlight the fact that we considered the change in valve properties as an isolated, constant disease. In reality, a valve pathology might also be related to a second disease, or the new grown state might trigger a remodeling process which can change tissue properties and the contractility of the myocardium, eventually leading toward heart failure. While more complex models can be used to better describe the disease, for the scope of this paper we did not consider this aspect crucial.

We describe cardiac growth only on the phenomenological level. Thus we assume that stress and strain can be sensed by the myocytes, but we neglect the underling process at the cellular level (Bellomo et al. 2012). We also neglect the long-term compensation mechanisms which might arise in response to the decrease in the hemodynamic function (Fig. 6).

Nevertheless, we think our results show how a simple left ventricular mechanics model is capable of describing in an elegant way the overall growth response to changes in global loading, taking into account the effect of growth on hemodynamics in a closed-loop circulation.

In conclusion, we investigated growth in a model coupling the LV mechanics at the tissue and organ level, that allows us to relate hemodynamics perturbations to a myofiber response, in terms of stress and strain. We observed that all four possible combinations of stress and strain stimuli with cavity and wall volume growth resulted into stable growth, albeit with different final cavity and wall volumes. Even if it is difficult to select the most appropriate stimulus-effect relation, most promising results were given by using at least one stress-based stimulus.

## Appendix

As described in Methods section, $\sigma _{\text {a}}$ depends on sarcomere length $l_{\text {s}}$, shortening velocity $v_{\text {s}}$ and the time elapsed from activation $t_{\text {a}}$. The constitutive law is written as (Bovendeerd et al. 2009):

11 $$\begin{aligned} \sigma _{\text {a}}\left( l_{\text {s}},\, t_{\text {a}},\, v_{\text {s}}\right) \;=\; f\left( l_{\text {s}}\right) \, g\left( t_{\text {a}},\, l_{\text {s}}\right) \, h\left( v_{\text {s}}\right) \end{aligned}$$

The dependence on $l_{\text {s}}$ is expressed as follows:

12 $$\begin{aligned} f\left( l_{\text {s}}\right) \;=\; {\left\{ \begin{array}{ll} \; 0 & l_{\text {s}} \le l_{\text {sa,0}} \\ \; \sigma _{\text {a,0}} \; \text {tanh}^{2}\left( c_{\text {a}}\left( l_{\text {s}} \;-\; l_{\text {sa,0}} \right) \right) & l_{\text {s}} > l_{\text {sa,0}} \end{array}\right. } \end{aligned}$$

where $\sigma _{\text {a,0}}$ is a constant active stress level, $l_{\text {sa,0}}$ identifies the length below which $\sigma _{\text {a}}$ is zero and $c_{\text {a}}$ defines the curvature behavior. The time dependence is modeled according to (van der Hout-van et al. 2012)

13 $$\begin{aligned} \begin{aligned} g\left( t_{\text {a}},\, l_{\text {s}}\right)&\;=\; {\left\{ \begin{array}{ll} \; 0 & t_{\text {a}} < 0 \\ \; \text {sin}^{2}\;\left( \frac{\pi }{2} \frac{t_{\text {a}}}{\tau _{\text {r}}}\right) & 0 \le t_{\text {a}} \le \tau _{\text {r}} \\ \; 1 - \text {sin}^{2}\; \left( \frac{\pi }{2} \frac{(t_{\text {a}} - \tau _{\text {r}})}{\tau _{\text {d}}} \right) & \tau _{\text {r}} \le t_{\text {a}} \le \tau _{\text {r}} + \tau _{\text {d}} \\ \; 0 & t_{\text {a}} > \tau _{\text {r}} + \tau _{\text {d}} \end{array}\right. }\\ \tau _{\text {r}}&\;=\; \tau _{\text {r,1}} + a_{\text {r}} \left( l_{\text {s}} - l_{\text {sa,1}}\right) \\ \tau _{\text {d}}&\;=\; \tau _{\text {d,1}} + a_{\text {d}} \left( l_{\text {s}} - l_{\text {sa,1}}\right) \end{aligned} \end{aligned}$$

Here the rise and decay time of the twitch are described by $\tau _{\text {r}}$ and $\tau _{\text {d}}$. For $l_{\text {s}} = l_{\text {sa,1}}$ the time constants are equal to $\tau _{\text {r,1}}$ and $\tau _{\text {d,1}}$, respectively. The evolution of $\tau _{\text {r}}$ and $\tau _{\text {d}}$ with sarcomere length is governed by parameters $a_{\text {r}}$ and $a_{\text {d}}$. Eventually the dependence on $v_{\text {s}}$ is modeled linearly:

14 $$\begin{aligned} h\left( v_{\text {s}}\right) \;=\; 1 - (v_{\text {s}} / v_{\text {0}}) \end{aligned}$$

where $v_{\text {0}}$ is the unloaded shortening velocity. All the parameter values of $\sigma _{\text {a}}$ (Table 1) are based on experimental data (Janssen and Hunter 1995; de Tombe and ter Keurs 1991). We consider the passive sarcomere stress as the wall stress generated by the collagen matrix along-fiber direction $\sigma _{\text {m,f}}$ and radial direction $\sigma _{\text {m,r}}$ (Bovendeerd et al. 2006). The first is defined as:

15 $$\begin{aligned} \sigma _{\text {m,f}}(\lambda _{\text {f}}) \;=\; {\left\{ \begin{array}{ll} \; \sigma _{\text {f,0}} \left[ \; \text {exp}\left( c_{\text {f}}\left( \lambda _{\text {f}} \;-\; 1 \right) \right) \;-\;1\;\right] & \lambda _{\text {f}} \ge 1 \\ \; 0 & \lambda _{\text {f}} < 1 \end{array}\right. } \end{aligned}$$

in which $\sigma _{\text {f,0}}$ is the constant passive stress level, while the along-fiber stretch $\lambda _{\text {f}}$ identifies the length below which $\sigma _{\text {m,f}}$ is zero. Eventually $c_{\text {f}}$ defines the curvature of the exponential behavior.

The passive transverse stress is modeled similarly:

16 $$\begin{aligned} \sigma _{\text {m,r}}(\lambda _{\text {r}}) \;=\; {\left\{ \begin{array}{ll} \; \sigma _{\text {r,0}} \left[ \; \text {exp}\left( c_{\text {r}}\left( \lambda _{\text {r}} \;-\; 1 \right) \right) \;-\;1\;\right] & \lambda _{\text {r}} \ge 1 \\ \; 0 & \lambda _{\text {r}} < 1 \end{array}\right. } \end{aligned}$$

In this case, we take in consideration the radial stretch $\lambda _{\text {r}}$. All the parameters shown for $\sigma _{\text {m,f}}$ and $\sigma _{\text {m,r}}$ (Table 1) are based on the pressure–volume relation of the passive LV taken from experimental data (Nikolić et al. 1988).

## References

[CR1] Arts T, Bovendeerd PHM, Prinzen FW, Reneman RS (1991). Relation between left ventricular cavity pressure and volume and systolic fiber stress and strain in the wall. Biophys J.

[CR2] Arts T, Delhaas T, Bovendeerd PHM, Verbeek X, Prinzen FW (2005). Adaptation to mechanical load determines shape and properties of heart and circulation: the circadapt model. Am J Physiol Heart Circ Physiol.

[CR3] Bellomo FJ, Armero F, Nallim LG, Oller S (2012). A constitutive model for tissue adaptation: necrosis and stress driven growth. Mech Res Commun.

[CR4] Bovendeerd PHM (2012). Modeling of cardiac growth and remodeling of myofiber orientation. J Biomech.

[CR5] Bovendeerd PHM, Borsje P, Arts T, van De Vosse FN (2006). Dependence of intramyocardial pressure and coronary flow on ventricular loading and contractility: a model study. Ann Biomed Eng.

[CR6] Bovendeerd PHM, Kroon W, Delhaas T (2009). Determinants of left ventricular shear strain. Am J Physiol Heart Circ Physiol.

[CR7] Buckert D, Cieslik M, Tibi R, Radermacher M, Rasche V, Bernhardt P, Hombach V, Rottbauer W, Wöhrle J (2018). Longitudinal strain assessed by cardiac magnetic resonance correlates to hemodynamic findings in patients with severe aortic stenosis and predicts positive remodeling after transcatheter aortic valve replacement. Clin Res Cardiol.

[CR8] Cantor EJF, Babick AP, Vasanji Z, Dhalla NS, Netticadan T (2005). A comparative serial echocardiographic analysis of cardiac structure and function in rats subjected to pressure or volume overload. J Mol Cell Cardiol.

[CR9] Carroll JD, Carroll EP, Feldman T, Ward DM, Lang RM, McGaughey D, Karp RB (1992). Sex-associated differences in left ventricular function in aortic stenosis of the elderly. Circulation.

[CR10] Cheng A, Langer F, Nguyen TC, Malinowski M, Ennis DB, Daughters GT, Ingels NB, Miller DC (2006). Transmural left ventricular shear strain alterations adjacent to and remote from infarcted myocardium. J Heart Valve Dis.

[CR11] Dewan S, Krishnamurthy A, Kole D, Conca G, Kerckhoffs RCP, Puchalski MD, Omens JH, Sun H, Nigam V, McCulloch AD (2017). Model of human fetal growth in hypoplastic left heart syndrome: reduced ventricular growth due to decreased ventricular filling and altered shape. Front Pediatr.

[CR32] de Tombe PP, ter Keurs HEDJ (1991). Sarcomere dynamics in cat cardiac trabeculae. Circ Res.

[CR12] Gaasch WH, Carroll JD, Levine HJ, Criscitiello MG (1983). Chronic aortic regurgitation: prognostic value of left ventricular end-systolic dimension and end-diastolic radius/thickness ratio. J Am Coll Cardiol.

[CR13] Göktepe S, Abilez OJ, Parker KK, Kuhl E (2010). A multiscale model for eccentric and concentric cardiac growth through sarcomerogenesis. J Theor Biol.

[CR14] Guzzetti E, Annabi MS, Ong G, Zenses AS, Dagenais F, Tastet L, Salaun E, Shen M, Piché ME, Poirier P, Voisine P, Pibarot P, Clavel MA (2019). Impact of metabolic syndrome and/or diabetes mellitus on left ventricular mass and remodeling in patients with aortic stenosis before and after aortic valve replacement. Am J Cardiol.

[CR16] Janssen PM, Hunter W (1995). Force, not sarcomere length, correlates with prolongation of isosarcometric contraction. Am J Physiol Heart Circ Physiol.

[CR17] Kainuma S, Taniguchi K, Toda K, Funatsu T, Kondoh H, Nishino M, Daimon T, Sawa Y (2011). Pulmonary hypertension predicts adverse cardiac events after restrictive mitral annuloplasty for severe functional mitral regurgitation. J Thorac Cardiovasc Surg.

[CR18] Kerckhoffs RCP, Omens JH, McCulloch AD (2012). A single strain-based growth law predicts concentric and eccentric cardiac growth during pressure and volume overload. Mech Res Commun.

[CR19] Kleaveland JP, Kussmaul WG, Vinciguerra T, Diters R, Carabello BA (1988). Volume overload hypertrophy in a closed-chest model of mitral regurgitation. Am J Physiol Heart Circ Physiol.

[CR20] Kroon W, Delhaas T, Arts T, Bovendeerd PHM (2009). Computational modeling of volumetric soft tissue growth: application to the cardiac left ventricle. Biomech Model Mechanobiol.

[CR21] Kusunose K, Cremer PC, Tsutsui RS, Grimm RA, Thomas JD, Griffin BP (2015). Regurgitant volume informs rate of progressive cardiac dysfunction in asymptomatic patients with chronic aortic or mitral regurgitation.. JACC Cardiovasc Imaging.

[CR22] Lamb HJ, Beyerbacht HP, de Roos A, van der Laarse A, Vliegen HW, Leujes F, Bax JJ, van der Wall EE (2002). Left ventricular remodeling early after aortic valve replacement: differential effects on diastolic function in aortic valve stenosis and aortic regurgitation. J Am Coll Cardiol.

[CR23] Nakano K, Swindle MM, Spinale F, Ishihara K, Kanazawa S, Smith A, Biederman RW, Clamp L, Hamada Y, Zile MR (1991). Depressed contractile function due to canine mitral regurgitation improves after correction of the volume overload. J Clin Investig.

[CR24] Nikolić S, Yellin EL, Tamura K, Vetter H, Tamura T, Meisner JS, Frater RWM (1988). Passive properties of canine left ventricle: diastolic stiffness and restoring forces. Circ Res.

[CR26] Rausch MK, Dam A, Göktepe S, Abilez OJ, Kuhl E (2011). Computational modeling of growth: systemic and pulmonary hypertension in the heart. Biomech Model Mechanobiol.

[CR27] Rawlins J, Bhan A, Sharma S (2009). Left ventricular hypertrophy in athletes. Eur J Echocardiogr.

[CR28] Roger VL, Seward JB, Bailey KR, Oh JK, Mullany CJ (1997). Aortic valve resistance in aortic stenosis: Doppler echocardiographic study and surgical correlation. Am Heart J.

[CR29] Seldrum Stephanie, de Meester Christophe, Pierard Sophie, Pasquet Agnes, Lazam Siham, Boulif Jamila, Vanoverschelde Jean-Louis, Gerber Bernhard L. (2019). Assessment of Left Ventricular Reverse Remodeling by Cardiac MRI in Patients Undergoing Repair Surgery for Severe Aortic or Mitral Regurgitation. Journal of Cardiothoracic and Vascular Anesthesia.

[CR30] Smith SH, Bishop SP (1985). Regional myocyte size in compensated right ventricular hypertrophy in the ferret. J Mol Cell Cardiol.

[CR31] Taber LA (1998). Biomechanical growth laws for muscle tissue. J Theor Biol.

[CR15] van der Hout-van MB, Oei SG, Bovendeerd PHM (2012). A mathematical model for simulation of early decelerations in the cardiotocogram during labor. Med Eng Phys.

[CR25] van Osta Nick, van der Donk Loes, Rondanina Emanuele, Bovendeerd Peter (2019). Modeling Cardiac Growth: An Alternative Approach. Functional Imaging and Modeling of the Heart.

[CR33] Villari B, Hess OM, Kaufmann P, Krogmann ON, Grimm J, Krayenbuehl HP (1992). Effect of aortic valve stenosis (pressure overload) and regurgitation (volume overload) on left ventricular systolic and diastolic function. Am J Cardiol.

[CR34] Weber KT, Brilla CG (1991). Pathological hypertrophy and cardiac interstitium. Fibros renin-angiotensin-aldosterone system. Circulation.

[CR35] Wisenbaugh T, Spann JF, Carabello BA (1984). Differences in myocardial performance and load between patients with similar amounts of chronic aortic versus chronic mitral regurgitation. J Am Coll Cardiol.

[CR36] Witzenburg CM, Holmes JW (2017). A comparison of phenomenologic growth laws for myocardial hypertrophy. J Elast.

[CR37] Witzenburg CM, Holmes JW (2018). Predicting the time course of ventricular dilation and thickening using a rapid compartmental model. J Cardiovasc Transl Res.

[CR38] Zile MR, Gaasch WH, Levine HJ (1985). Left ventricular stress-dimension-shortening relations before and after correction of chronic aortic and mitral regurgitation. Am J Cardiol.

[CR39] Zile MR, Tomita M, Nakano K, Mirsky I, Usher B, Lindroth J, Carabello B (1991). Effects of left ventricular volume overload produced by mitral regurgitation on diastolic function. Am J Physiol Heart Circ Physiol.

